# From policy to plate: stakeholder perspectives on nutrition policy in Australian early childhood education environments

**DOI:** 10.1093/heapro/daaf165

**Published:** 2025-10-03

**Authors:** Anna Aristova, Alison C Spence, Christopher Irwin, Penelope Love

**Affiliations:** Institute for Physical Activity and Nutrition (IPAN), School of Exercise and Nutrition Sciences, Deakin University, Waurn Ponds, VIC, Australia; Institute for Physical Activity and Nutrition (IPAN), School of Exercise and Nutrition Sciences, Deakin University, Waurn Ponds, VIC, Australia; School of Allied Health, Sport and Social Work (SHS), Nutrition and Dietetics, Griffith University, Southport, Gold Coast, QLD, Australia; Institute for Physical Activity and Nutrition (IPAN), School of Exercise and Nutrition Sciences, Deakin University, Waurn Ponds, VIC, Australia

**Keywords:** early childhood education, pre-school, nursery, nutrition policy, childcare

## Abstract

Early childhood education and care (ECEC) settings are pivotal in shaping children's dietary behaviours. While the importance of centre-based nutrition policies (CBNPs) in shaping early childhood nutrition environments is well established, little is known about how these policies are interpreted and implemented by those working in the sector. In particular, limited research has explored the perspectives of both service-level (ECEC employees) and agency-level (health/government organization) stakeholders, which are two groups central to supporting and enacting these policies in practice. This study used a qualitative exploratory design to understand stakeholder experiences and perspectives regarding the implementation of CBNPs and the broader challenges in fostering supportive ECEC nutrition environments. Semi-structured Zoom interviews were conducted with 9 ECEC employees and 10 agency-level representatives across 8 Australian jurisdictions. Interview data were analysed using reflexive thematic analysis. Three overarching themes were identified, reflecting how stakeholders understood the importance of CBNPs and what factors they believed were necessary for successful implementation: (i) the need for realistic rather than idealistic policy requirements, (ii) adaptation as a prerequisite for implementation, and (iii) the value of a multi-faceted approach in creating optimal nutrition environments. Findings point to the need for a multi-faceted approach that combines adaptable guidelines with targeted, practical support such as training and resources, tailored to the realities of ECEC settings. Strengthening partnerships between policymakers, educators, families, and health professionals is critical to supporting the sector and developing feasible strategies that enhance CBNP implementation and promote healthy eating among young children.

Contribution to Health Promotion

- Highlights how early childhood settings support children’s health through nutrition policies and practices
- Shares insights from ECEC employees and health professionals about real-world challenges in promoting healthy eating
- Shows the gap between nutrition guidelines and the resources/services needed to put them into action
- Emphasizes the importance of staff training, family engagement, and government support in improving children’s nutrition environments
- Provides recommendations to make nutrition policies more practical, realistic, and supportive for early childhood settings

## INTRODUCTION

Nutrition in early childhood, from birth to age 5, is pivotal to growth, development, and overall health (Mameli *et al*. 2016). Establishing healthy dietary patterns during this formative period can have lasting effects, including a reduced risk for developing chronic diseases and unhealthy weight status later in life (Afshin *et al*. 2019, Wood *et al*. 2020). Recognizing this, public health efforts have increasingly focused on early childhood nutrition promotion as an important approach to mitigate the long-term burden of diet-related disorders across the lifespan (Howe *et al*. 2015, Wood *et al*. 2020, Kashef *et al*. 2023, Morgillo *et al*. 2024, Elford *et al*. 2025).

Across high-income countries, early childhood education and care (ECEC) services play a significant role in the daily lives of young children, with high enrolment rates reflecting their widespread use (Jackson *et al*. 2021). In Australia, ECEC service use continues to increase, particularly long day-care centres (LDCs; also referred to as centre-based day care or childcare), with ∼60% of Australian children 0–5 attending these facilities (Australian Government Department of Education 2023). According to the most recent national data, over 9000 approved LDC centres currently operate across the country (Australian Children’s Education and Care Quality Authority 2024). The delivery of LDC service in Australia occurs through a mix of private for-profit providers, not-for-profit organizations, and social enterprises, each shaped by distinct structures, resources, and operational priorities. LDC centres operate for extended hours (minimum 8 h/day and 48 weeks/year), and those services that include food provide a minimum of two snacks and one main meal per day (Australian Government Department of Health 2015). Food provision models vary, with some centres employing a cook to prepare meals on-site, while others require families to send packed food from home (i.e. lunchbox services). Australian children attending LDCs spend an average of 27 h per week in such settings, accounting for a substantial portion of their weekly dietary intake (Australian Government Department of Education 2023). There remains a significant gap between the dietary intakes of children in LDC settings, and the recommendations outlined in national nutritional guidelines (New South Wales Ministry of Health 2025, National Health and Medical Research Council [NHMRC] 2013, Yoong *et al*. 2014, Jones *et al*. 2017). Research in Australia and internationally has consistently shown that children in these settings often have inadequate intakes of essential food groups, such as vegetables, fruits, and whole grains, while overconsuming foods high in saturated fats and sugars (Frampton *et al*. 2014, Yoong *et al*. 2014, Dixon *et al*. 2016, Jones *et al*. 2017, Grady *et al*. 2020, Sambell *et al*. 2020, Hasnin *et al*. 2022).

The operational framework for Australian ECEC services is governed by the National Quality Framework (NQF), with the Australian Children’s Education and Care Quality Authority (ACECQA) overseeing compliance with the National Quality Standards (NQS; Australian Children’s Education & Care Quality Authority National Quality Framework [ACECQA]). Under the NQS, services are assessed and rated across seven quality areas, with nutrition addressed under Quality Area 2: Children’s Health and Safety. Licensing and accreditation processes require services to demonstrate compliance with standards through documentation, observations, and evidence aligned with ACECQA’s assessment and rating instrument, including supporting questions specific to food provision and healthy eating practices. In line with this framework, ECEC services are required to have policies and procedures in place that address nutrition, food, beverages, and dietary requirements to ensure children receive nutritionally appropriate meals. These regulatory expectations provide an important foundation for the development and implementation of centre-based policies (CBNP), setting a national benchmark for the promotion of healthy eating within early childhood settings.

In Australia, CBNPs are designed to assist ECEC services in fostering an optimal nutrition environment that promotes the health and well-being of children. Similar policies exist internationally under various terms, such as childcare food policies, wellness policies, or early childhood nutrition standards (Cotwright *et al*. 2017, Hotz and Wiswall 2019, Andreyeva *et al*. 2021). These policies, intended to be individualized to each service, aim to ensure that the food provided aligns with national dietary guidelines and meets children's nutritional needs. CBNPs typically cover areas such as food provision, nutrition education, and strategies to engage families in promoting balanced eating habits and, in some cases, may outline specific nutrient targets. While some international tools and standards assess the inclusion of nutrient-specific targets, Australian CBNPs do not typically require this level of detail. However, given the diversity in policy content and limited national data on their scope, it is plausible that some services may include such targets. In most cases, alignment is achieved through reference to recommended food group intakes or use of menu planning tools. Beyond guiding food provision, CBNPs can also shape the broader food environment by promoting positive feeding practices and modelling healthy eating behaviours during daily routines (Byrne *et al*. 2022, Kirkegaard *et al*. 2024).

Although Australia does not have specific national nutrition guidelines tailored solely to LDC, it does have multiple levels of guidance. National guidelines such as the Australian Dietary Guidelines inform a range of ECEC-specific resources and programmes, such as Get Up and Grow and Caring for Children, which are widely used across jurisdictions (i.e. individual states and territories). These, alongside guidance from ACECQA, which oversees the NQF but does not provide direct nutrition-specific support, form part of a broader suite of resources that assist LDC services in developing and implementing CBNPs (Australian Government Department of Health 2015). Additional support is also available through state-based initiatives such as Munch and Move (Healthy Kids NSW Department of Education and Training 2020) or the Healthy Eating Advisory Service (HEAS) 2025, as well as non-government organizations, state and territory health departments, and ECEC-specific training programmes. These may include menu planning tools, nutrition training programmes, and professional development opportunities for ECEC employees to help services align with dietary guidelines. However, access to these resources may vary, and the extent to which they effectively support policy implementation remains an important consideration (Spence *et al*. 2020).

However, the content and implementation of these policies vary across settings, influenced by factors such as policy quality, organizational culture, resource availability, and staff training (Jones *et al*. 2015, Liu *et al*. 2016, Wolfenden *et al*. 2020, Aristova *et al*. 2024). Ensuring that these policies are well designed and effectively implemented remains challenging. Research indicates that ECEC services face barriers such as limited resources, varying levels of staff knowledge and confidence in nutrition, and competing priorities, which can hinder the development and consistent implementation of an effective CBNP (Cumming 2017, Wallace *et al*. 2017, Thorpe *et al*. 2021).

In addition to service-level efforts, the success of CBNPs is also influenced by external stakeholders, such as health organizations and government agencies, who play an important role in shaping the ECEC nutrition policy landscape (Green *et al*. 2020, Jackson *et al*. 2021). These agency-level stakeholders, across the different jurisdictions, can be instrumental in developing guidelines, providing resources, and promoting health initiatives within ECEC environments (Green *et al*. 2020, Jackson *et al*. 2021). Their perspectives, alongside those of ECEC employees, are therefore critical to understand how CBNPs are enacted and the challenges faced by the ECEC sector in translating such policy into practice. Equally important are the insights of ECEC employees, who are directly responsible for interpreting and implementing these policies on the ground. Their experiences offer valuable context on the day-to-day realities of policy enactment, including practical constraints and opportunities for improvement, while also highlighting their active role in shaping, implementing and co-designing effective nutrition policies.

This study sought to answer the following research questions: (i) How do service-level (ECEC employees) and agency-level (relevant external health organizations) stakeholders perceive the role and implementation of centre-based nutrition policies (CBNPs) in early childhood settings and (ii) What factors influence effective implementation of CBNPs and the creation of supportive nutrition environments in ECEC settings? Understanding stakeholder experiences and perspectives will support identification of barriers and enablers to effective CBNP implementation and opportunities for improvement, to better enable ECEC services to support healthy eating habits of children.

## MATERIALS AND METHODS

### Study design

This study presents findings from qualitative interviews conducted as part of a broader study examining CBNPs in ECEC settings, which included an online survey and follow-up semi-structured interviews. While the interviews followed the survey, they were conducted to explore broader stakeholder perspectives, particularly regarding policy implementation and nutrition environments. The survey focused on policy content and will be reported separately. Demographic data collected via the survey is included here to contextualize the interview sample. Qualitative interviews explored stakeholder experiences, perceptions, and challenges relating to the implementation and effectiveness of CBNPs as well as broader factors considered influential to creating a supportive nutrition environment in ECEC settings.

### Recruitment

Participants were targeted to capture both internal and external perspectives on nutrition policy and implementation. Service-level stakeholders provided insight into the practical realities of working within services, while agency-level stakeholders offered a broader, system-level understanding based on their roles in supporting, guiding, or regulating early childhood nutrition environments.

For the ECEC service-level interviewee group, survey invitations were emailed to the publicly listed contact addresses of all Australian LDC centres available on the ACECQA website (*n* = 12 268). These invitations were broadly addressed to ECEC employees, including educators, cooks/chefs, and directors, and provided details about the study along with a direct link to the survey. While emails were sent to the centre’s general contact address, individual employees were able to self-select into the study upon receiving the information. Eligibility was restricted to those currently employed at an LDC centre in Australia. At the end of the survey, participants could indicate if they were willing to participate in a follow-up interview.

For the agency-level interviewee group, a comprehensive online website search identified potential organizations using terms such as ‘Australian health organisations’, ‘Australian nutrition organisations’, ‘Australian childcare health’, ‘Early childhood nutrition’, ‘Childcare nutrition guidelines Australia’. The resulting list was reviewed and approved by the research team, and invitations were sent to relevant Australian health and government organizations (*n* = 31). As with the ECEC group, survey participants were given the option to express interest in a follow-up interview.

### Data collection

Demographic data, including role, jurisdiction, and nutrition training, were collected through the online survey and are reported in this paper to provide context for interview participants. A semi-structured interview protocol (Supplementary File S1) was developed, reviewed, and approved by the research team and pilot tested to ensure clarity and logical flow. Interview questions explored stakeholder roles, experiences with nutrition policy development and implementation, challenges and support needs in ECEC settings. Where relevant, questions were tailored to reflect participants’ survey responses to allow for deeper exploration of their perspectives. All individuals who consented to an interview were contacted by the lead researcher (A.A.) who scheduled and conducted all interviews via Zoom (version 5.17). All individuals who consented to participate were interviewed. Recruitment continued until no further responses were received, and by the final interviews, no substantially new information was emerging, suggesting that key themes had been adequately explored. Interviews lasted ∼1 h and were recorded with participant consent. Zoom’s automatic transcription function was used, and transcripts were checked for accuracy against recordings by A.A. The interviews explored stakeholder experiences and perspectives relating to the implementation and effectiveness of CBNPs in ECEC settings and broader challenges and enablers in fostering supportive nutrition environments.

### Ethical approval

This study was conducted in accordance with the Declaration of Helsinki and received approval from the Deakin University Human Ethics Advisory Group (approval no. HEAG-H 34_2023). All participants provided informed written consent.

### Data analysis

Data were analysed using Braun and Clarke’s reflexive thematic analysis (RTA), following their six-phase process: familiarization, coding, theme development, review, definition, and final write-up. Reflexivity was an important consideration throughout the research process (O’Brien *et al*. 2014). The lead author (A.A.) is a PhD candidate in public health with a focus on nutrition policy in ECEC settings. All co-authors, P.L., A.C.S. and C.I., are dietitians with academic roles teaching and researching in public health nutrition and community dietetics. P.L. and A.C.S. have engaged with ECEC settings as parents. The collective expertise and experience of the research team informed the interpretation of the data, with a shared intent to generate meaningful insights that can inform future strategies for improving nutrition policy and practice in ECEC settings.

Demographic data collected during the survey phase were summarized using descriptive statistics. Online interviews were transcribed using Zoom software v5 and then imported into NVivo v14 for qualitative analysis. RTA was employed to explore patterns and themes related to the ECEC nutrition environment following the six-step process recommended by Braun and Clarke (2022).

Familiarization involved immersion with the data through repeated reading of transcripts to identify meaningful patterns. An inductive, iterative coding process was then undertaken by A.A., with codes refined reflexively with the research team to inform theme development. Initial codes were generated through an inductive approach, with A.A. systematically coding significant features of the data. This coding process was reflexive and iterative, with codes evolving in response to developing insights and continued engagement with the data. To account for the two stakeholder groups (ECEC service level and agency level), data were initially coded without differentiation, allowing candidate themes to develop naturally from the entire dataset. Responses were then examined separately by stakeholder group to determine whether perspectives aligned or differed across codes. This approach ensured that both shared and contrasting viewpoints were captured while maintaining a consistent analytical process. The third step involved searching for patterns by collating the codes into candidate themes and identifying relationships between them. Candidate themes were then reviewed to ensure that they accurately captured the meaning of the data and were coherent both within and across themes and further refined and defined to ensure they provided a detailed and nuanced understanding of the ECEC nutrition environment. Finally, vivid and compelling examples from the data were selected to illustrate the themes. Themes were reviewed by the entire research team at multiple stages of the analysis process, including after initial coding and during final theme definition. Reflexivity was maintained through ongoing journalling, discussion, and acknowledgement of researcher subjectivity throughout theme development and interpretation. To support consistency, P.L. reviewed a subset of transcripts during initial coding and theme refinement. Differences were discussed and coding adjusted, ensuring a robust interpretation of the data.

## RESULTS

A total of 133 service-level ECEC employees completed the survey, 33 consented to a follow-up interview and all were contacted, with 9 interviews conducted. For the agency-level group, 17 stakeholders completed the survey, 12 consented to a follow-up interview, and all were contacted, with 10 interviews conducted. While 45 participants across both groups consented to a follow-up interview, only 19 interviews were conducted due to non-responsiveness to follow-up scheduling emails. A maximum of two reminders were sent to each consenting participant, as permitted under the study’s ethics approval. Participants represented a diverse range of roles and were drawn from across all Australian states and territories (Table 1). Agency-level stakeholders worked in senior or advisory capacities across a mix of government and non-government organizations involved in early childhood health promotion and nutrition policy support (Table 1). For the service-level group, most participants were based in two jurisdictions (Victoria and New South Wales) and included directors, educators, and chefs, with varied levels of experience in their roles. While all nine participating centres reported having on-site food provision, two also responded that they have the option for families to bring food from home, resulting in a hybrid approach to food provision (Table 2).

**Table 1.** Demographic characteristics of agency-level stakeholders.

Characteristic	Agency-level stakeholders (%) (*n* = 10)
Jurisdiction	
VIC	30
NSW	10
QLD	0
SA	10
WA	10
TAS	20
ACT	10
NT	10
Qualifications^a^	
Nutrition/dietetics	91
Psychology	9
Medicine	9
Education	9
Other	18
Organization type	
Government (state/territory)	40
NGO or not-for-profit organization	60

ACT, Australian Capital Territory; NSW, New South Wales; NT, Northern Territory; QLD, Queensland; SA, South Australia; TAS, Tasmania; VIC, Victoria; WA, Western Australia.

^a^Percentages for agency-level stakeholder qualifications exceed 100% as some participants reported holding multiple qualifications.

**Table 2.** Demographic characteristics of service-level stakeholders.

Characteristic	Service-level stakeholders (%) (*n* = 9)
Jurisdiction	
VIC	44
NSW	44
QLD	11
SA	0
WA	0
TAS	0
ACT	0
NT	0
Role	
Director	56
Educator	33
Chef	22
Length of time in role, years	
<1	11
1–5	11
5–10	33
>10	44
Works in a centre where food is prepared	
Onsite	100
From home	22
Nutrition training	
Yes	22
No	78

ACT, Australian Capital Territory; NSW, New South Wales; NT, Northern Territory; QLD, Queensland; SA, South Australia; TAS, Tasmania; VIC, Victoria; WA, Western Australia.

### Perspectives on centre-based nutrition policies

Participants consistently emphasized the vital role of CBNPs in shaping nutrition environments within ECEC settings. Most recognized these policies as fundamental to promoting healthy eating habits among children and maintaining nutrition standards. However, they also identified key challenges and considerations as essential to enhance CBNP effectiveness. Three overarching themes were identified, reflecting nuanced perspectives: (i) balancing realistic and idealistic policy expectations, (ii) adaptation as a prerequisite for implementation, and (iii) recognizing that a multi-faceted approach supports optimal nutrition environments. These themes are explored through interrelated subthemes that reflect specific issues and stakeholder insights. Hereafter, the term ‘policy’ refers to CBNPs.

### Theme 1: balancing realistic and idealistic policy requirements

Both participant groups emphasized the importance of implementing nutrition policies and practices that are realistic and achievable within the constraints of ECEC settings. While the need for best practice goals was recognized, participants, particularly service level, frequently highlighted the challenges in adhering to strict and prescriptive standards due to resource limitations, varying levels of staff expertise, and the practicalities of daily operations. The call for realism reflected an understanding that ambitious nutrition goals, though well intentioned, must coexist with the fast-paced and unpredictable nature of ECEC environments.

#### Policy consistency

Both participant groups highlighted that a well-structured CBNP helped reinforce consistency within ECEC services by ensuring that staff and parents adhered to the same nutrition principles. Policies were viewed as safeguarding against inconsistent practices by reducing the influence of individual discretion.

It doesn't allow for personal opinion to come in. There's sometimes 22 of us working and one of us might let something slide, and the other one might not. I think if you've got that in policy, it protects the children and ourselves. There's no room for personal opinions in it. (ECEC, Director)

It's a place where it keeps things consistent in the service (Agency, Public Health Dietitian)

Consistency was seen as a protective factor for both children and staff, ensuring that nutrition-related decisions were aligned with shared expectations. Policies were also described as the foundational structure that united staff and families:

It's the backbone of the service. For food and nutrition, having that [policy] in place sets the tone, and then nobody can argue with it because staff have signed it and so have parents…we're all promoting that actively going forward. (ECEC, Director)

These accounts suggest that strong policies function not just as guidelines, but as institutional anchors that foster collective accountability and reduce ambiguity in decision-making.

#### Policy prioritization

A significant challenge expressed by service-level participants to implementing CBNPs was the prioritization of daily tasks over policy adherence, with operational demands often overshadowing policy intentions in everyday practice. Service-level participants noted that, despite understanding CBNPs, the high demands of their roles frequently pushed nutrition-related tasks to the background.

On a day-to-day basis, you don't know the policy exists because no one talks about it, in a meeting no one highlights it. The most important part of everyday operation goes to planning and programming how to deal with challenging behaviour (ECEC, Educator)

I noticed that generally all educators are educated about what’s required. The main problem is it’s so busy sometimes that you don’t have time to follow that voice. Lunchtime in childcare is literally the busiest time I ever experienced in my life. We’re trying to follow that policy, but sometimes because it’s so busy and demanding, you don't have time. Sometimes you know what’s right to do, but you can't do it. (ECEC, Educator)

These perspectives reveal a disconnect between policy understanding and policy enactment, where knowledge of best practices is overridden by the intensity of the ECEC environment.

Agency-level participants expanded on this challenge, stressing the need for fidelity checks to ensure implementation was not just assumed but assessed:

[Fidelity checks] should be mandatory because we're putting kids at risk. I think [fidelity checks] would realistically be a huge challenge but if you're going to have standards and policies, you need to check up on them because people can make all sorts of claims, and they're not necessarily true. (Agency, Research Scientist)

Fidelity is framed here not just as a technical quality assurance measure, but as a mechanism to safeguard children by ensuring that written policies translate into actual practice.

#### Policy language

Both participant groups also stressed the importance of developing CBNPs using accessible and user-friendly language. Agency-level participants emphasized the need to strike a balance in policy specificity to ensure policies are detailed enough to be meaningful, yet not so complex that they become difficult for ECEC employees to interpret and implement effectively. Clarity and accessibility were seen as essential to enhancing policy uptake, with participants noting that overly technical language can reduce engagement and disconnect policy from practice.

[Nutrition] policy documents themselves are too long. They’re too complex, and they’re not written in a language that end users can easily understand and apply. (Agency, Government-funded service representative 2)

The more specifics you can put in the better of course, from a nutrition perspective. But there's no sense in having something that people can't understand. [ECEC centres] do have policies but it's sitting on a shelf and it's got no relationship to what is actually being done day-to-day on the ground. (Agency, Public Health Dietitian)

This concern was echoed by service-level participants, who highlighted that policies lacking clarity or specific guidance often failed to engage ECEC educators meaningfully.

My centre policy was very generic. It doesn’t give us those dot points… there wasn’t enough information strong enough. That’s probably why I’ve never looked at it again… If it was very specifically written, then it would add to my knowledge… But it wasn’t. The language was not in a form that it should be. (ECEC, Educator)

The cumulative message was clear in that the utility of a policy depends on its content and also on its ability to be understood, trusted, and applied by those working under its guidance.

#### Policy focus

Both agency-level and service-level participants voiced concerns regarding policies containing overly prescriptive nutrient content limits and nutrition standards. They emphasized that while meeting children's nutritional needs is paramount, overly rigid guidelines may be unrealistic and could detract from what some viewed as the primary goal of ensuring children consume adequate food. Both groups stressed the importance of clear, achievable, and realistic nutrition guidelines rather than overly prescriptive ones.

When you have all that going on, no one cares about the salt in food, you just want this child to eat something, stop crying and get them ready to sleep and know they have at least had something to eat. (ECEC, Educator)

It needs to be practical when considering these [standards], because being too prescriptive, can cause great difficulties Going down into the minutia of how many grams of sodium we're going to allow in a particular dish is not practical. To prescribe the milligram content of sodium in a recipe, it sets everyone up to fail, it makes it too hard. It makes it too expensive to assess as well. (Agency, Senior Dietitian)

These concerns demonstrate how nutrition goals perceived as ‘gold standard’ may clash with frontline realities. Rigid standards were seen as burdensome, potentially undermining efforts to meet children’s basic food needs in high-pressure environments.

Collectively, these perspectives expressed the need for policies that set clear nutrition goals without compromising feasibility, cost, or educator capacity. Clarity, prioritization, and consistency were not just implementation facilitators but essential levers for reconciling idealistic goals with everyday realities.

### Theme 2: adaptation as a prerequisite for implementation

Another key theme identified was the need to contextualize nutrition guidelines in policies to reflect the diverse realities of ECEC services. Both participant groups noted that a one-size-fits-all approach often falls short in meeting the diverse needs of services, which vary widely in terms of demographics, cultural contexts, geographic location, and available resources. While national- and state-level nutrition guidelines provide a useful starting point, participants across both groups described how rigid application of these standards was often unfeasible. Instead, they called for CBNPs that are flexible and responsive to their specific service environment. This theme highlights that implementation is unlikely without adaptation and that policy effectiveness depends on how well it accommodates the practical and social realities of each ECEC setting.

#### Flexibility to adapt standards

Both participant groups emphasized the importance of contextualizing national and state healthy eating guidelines through CBNPs in ECEC settings. Agency-level participants highlighted that while guidelines offer valuable direction, they are not always easily applicable across diverse service contexts. Several service-level participants expressed concern that aspects of these guidelines can feel prescriptive in practice, particularly when services are expected to meet ideal nutrition benchmarks without adequate flexibility or support. Service-level participants echoed the need for discretion in adapting guidelines to reflect the cultural, operational, and nutritional needs of their communities. Findings indicate that policies are more likely to be implemented when they support local interpretation rather than impose rigid, top-down requirements.

…because no one considers what happens in a regional community where there’s low socioeconomic areas. They don’t have regular access to fresh food, yet we’re saying they need two fruits, veg, all these things on the menu each week. They can’t achieve that. So what is the contextualised solution for them? (Agency, Government-funded service representative 1)

…that could be at the discretion of centres to work out how much they serve. As long as they're implementing those responsive feeding principles—in that children can choose not to eat the whole amount, ask for more or eat until they feel full. (Agency, Research Fellow)

#### Responding to food insecurity

Both participant groups also highlighted that effective implementation requires policy to reflect the socioeconomic conditions of the families and communities they serve. In particular, service-level participants described how food insecurity shaped children’s eating behaviours and nutritional needs in ways that rigid nutrition standards often failed to accommodate. Unlike concerns about food costs within ECEC services, discussion focused on the broader financial pressures faced by families and how these constraints impact children's access to food, both at home and within ECEC.

Some of our children will eat bowls and bowls and bowls because they worry that it's going to be their last, they know they're not going to get dinner. So knowing that, when do we stop them to teach them when they're full but also give them enough so they know when they go home they'll be okay? (ECEC, Director)

This highlights how some children’s lived experiences directly contradict the assumptions underpinning nutrition guidelines, particularly those that presume stable food access and consistent meal patterns.

#### Cost-related constraints on implementation

Beyond the need for policy flexibility and responsiveness to family circumstances, service-level participants also identified structural barriers to implementing nutrition standards, particularly rising food costs. While they explained they understood they are expected to adhere to nutrition guidelines, rising food prices often limited their ability to provide meals that met these standards. Food cost pressures influence menu planning and ingredient selection which affected CBNP implementation. In some cases, service-level stakeholders reported having to reduce the nutritional quality of meals to remain financially viable, while others described making sacrifices in other areas to prioritize food quality. These experiences reinforce that even well-designed policies can falter if they are misaligned with the economic conditions under which services operate.

You have to make cuts and sometimes it’s to the detriment of the children, because things like proteins and vegetables are costly, so it’s cheaper to bulk it out with pasta and rice than it is to make sure they’re meeting those needs. (ECEC, Educator and Cook)

Within that budget you must include morning teas, afternoon teas, everything. Even if families suggest adding more meat, dry fruits or high-quality cheeses, it’s not feasible because it will be expensive. This is pulling childcare down because of cost and budget. (ECEC, Educator)

We have lamb cutlets which costs us an arm and a leg to feed 70 children. They're small, low fat lamb cutlets. The 4-year-olds are having 2 of those and it costs us. It's a huge budget for us but they love it. They sit there and they eat it down to the bone, they just love lamb cutlets and veggies day, wouldn't have it any other way. You just make cuts elsewhere to make that happen. (ECEC, Director)

These insights reinforce that effective policy implementation depends on the capacity to adapt guidelines to the practical, cultural, and economic conditions faced by individual services.

### Theme 3: multi-faceted approach

Both participant groups consistently stressed the need for a multi-faceted and coordinated approach to create optimal nutrition environments in ECEC settings. This involved integrating CBNPs with broader health promotion strategies, including staff training, family engagement, and collaboration with external health agencies. This system-based approach was seen essential for achieving consistent and sustainable improvements in nutrition environments.

#### Family engagement

Both participant groups emphasized the value of building strong partnerships with families to reinforce healthy eating habits both within and beyond the service. Service-level participants further noted that educating families and maintaining open, supportive communication led to improved food choices and greater alignment with nutrition policy goals.

I've never realised how important relationships with families and food was and how beneficial it could be. I saw first-hand how much better food choices became, the more we educated [parents] (ECEC, Director)

#### Child engagement

Many service-level and agency-level participants emphasized the importance of diverse food-related activities for educating children about nutrition within ECEC services, suggesting that such initiatives could be more explicitly reflected in CBNPs, such as edible (food) gardens, cooking classes, and sensory exploration to provide hands-on experiences that promote healthy eating habits. Both groups favoured a move beyond traditional nutrient-focused education to discussions that explored food origins and environmental stewardship, fostering curiosity and a deeper appreciation for food among children while supporting a holistic strategy for nutrition education.

We haven't managed to get much of the plant into the kitchen because kids are out there eating it. I've put snow peas on a platter, and they won’t touch them, but then they'll eat them directly off the plant. That education of you planted it, you grew it, you've been watering it, now reap the benefit of it. (ECEC, Director)

They're too young to need to learn, they can have basic understanding of energy but that's it. To teach them stuff about nutrition at that age… it's more about the exposures, positive experiences and engagement with food like doing some foodie activity like making sandwich sushi. (Agency, Senior Dietitian)

#### Service-level recognition

Several service-level participants described a desire for external recognition of their efforts in implementing CBNPs. They noted that formal acknowledgement from agencies, such as certifications or assessments, could serve as an incentive for services to maintain nutrition standards. They highlighted that such recognition could enhance morale among employees and foster a positive atmosphere within the ECEC setting.

I'd love there to be a certification or assessment you can do to get recognised for all the hard work. I think for services exceeding an excellent level, being able to stick something on the front door that says we’re certified by […] we want that logo on our front door. Then it means we must keep this standard up. (ECEC, Director)

#### Cross-role collaboration

Service-level participants described that effective nutrition practices relied on meaningful whole-of-service collaboration across all roles. While ECEC educators and directors are typically involved in implementing CBNPs, participants noted that ECEC cooks/chefs were frequently left out of staff meetings or professional development activities despite their critical role in food provision decision-making processes. Participants expressed that this lack of integration was seen to limit opportunities for shared understanding and undermined cohesive nutrition practices.

…make the menus nutritious as possible, work with chefs to increase their knowledge of nutrition and work alongside management to make sure that we’ve actually got the policies and procedures in place to back what we’re saying. (ECEC, Director)

With staff meetings, chefs won’t be involved and I think the involvement of chefs is important, even though it’s just the kitchen, you’re in the education sector right? If you’re part of meetings, you’d start thinking critically about food and understand children’s needs. The chef should be involved in meetings. Their response and voice should be equally heard and acknowledged. (ECEC, Educator)

Both groups further advocated for training modules and professional development that included all ECEC employees, including kitchen personnel, to ensure consistent nutrition knowledge and practice across roles. Educators reported that limited nutrition training hindered their ability to engage with other staff on food-related matters, while agency stakeholders emphasized the need for structured systems that embed foundational training and ongoing professional development.

We have food handling and food safety training, but the nutrition part isn’t there. It would have helped us—because sometimes we feel like we don’t have enough knowledge to talk about it to our chef or managers. A short training or module would be really helpful. (ECEC, Educator)

Cooks feel displaced in services… needs to be more interrelated within the service. Policy can include professional development, but I think a better assistance approach is to develop a certificate course for cooks so they have this foundational knowledge, and then it’s a matter of providing refreshers within their professional development. (Agency, Government-funded service representative 1)

#### Implementation support

Many participants from both groups reported concerns regarding the adequacy of support available to ECEC services for the development and implementation of CBNPs. They highlighted that the current support mechanisms, which include training workshops, nutrition resources, and professional development, often fall short in meeting the needs of ECEC services, making the process cumbersome. Participants noted that disparities exist in the availability of implementation support across jurisdictions and service types, which can affect the successful implementation of CBNPs.

Having more resource, trainings, and a template to follow would be really good, especially not knowing all the nutrients and having one million things to do. It would be nice if there were already resources with a link to click and I just read it, I think that's what I would be needing. (ECEC, Educator)

…then you've got a director and cook working together, especially if there's literacy barriers, the time it takes back and forth with email or phone, sometimes I've gone out and spent 6 hours sitting in a director's office going through and making the menu up to scratch. (Agency, Public Health Dietitian)

Agency-level participants also identified equity concerns, highlighting that current system may inadvertently favour services with greater capacity or resourcing, potentially widening existing gaps.

…because [our agency’s role is] a state-wide thing, you end up spending heaps of time with services that have a bit more money to get their thing perfect, and then they're back the next year with another menu that they want to review so [the current system] doesn't check the equity box. (Agency, Public Health Dietitian)

#### Government support

Most participants from both groups expressed a strong need for more coordinated and accessible government support to improve nutrition practices and strengthen implementation of CBNPs. This included calls for clearer and more accessible resources, as the current landscape of nutrition regulations and guidance was described as fragmented and difficult to navigate.

One of the problems with the government is they give us these regulations, but then we have to go to all different places to find it. (ECEC, Director)

Both groups also advocated for mandatory nutrition training, noting that many employees are expected to navigate nutrition responsibilities without adequate knowledge or support.

[Centres] have mandatory requirements but not a lot of nutrition. They’re kind of expected to know or go off a nutrition policy. But working in the ECE space, it's so important to see the value in education for all ECEC employees (Agency, Senior Dietitian)

In addition to resourcing and training, both groups stressed the importance of increased government funding to address core operational challenges, particularly food quality and affordability. Service-level participants highlighted that without additional financial support, many services remain limited in their capacity to provide nutritious, balanced meals, often relying on the cheapest available ingredients. Affordable, high-quality food provision was described as a priority that requires stronger government investment.

The budgets need to be increased. I know some of the big centre costs are a dollar per head, which means you can only cook kids pasta and beans on toast, because there isn't enough money. Vegetables and proteins cost more, but they cost more for a reason, they’re for the benefit of the children. And that nutrition is important. And I'm advocating for that. (ECEC, Chef & Educator)

Programmes such as ‘Munch & Move’ (in NSW), and the ‘Achievement Program’ (in Victoria) were viewed as helpful starting points for promoting healthy practices and encouraging a whole-of-service approach to health promotion. However, participants noted that the availability, intensity, and level of support for these initiatives varied across jurisdictions.

Munch & Move is a whole different level—they’ve got a full program and a dedicated workforce across the state so there’s much higher adoption, it’s a lot more intensive. In Victoria, it works differently. We’re here to support the long daycares and health promotion officers but with resources we have. Other states don’t have a workforce at all. Still it’s a government priority—at least it’s on the agenda. (Agency, Program Manager)

We’re part of the Healthy Achievement Program, which includes nutrition and wellbeing. We lost our way with it over COVID… but we’ve dug back into it the last 18 months. That’s why we’ve looked at our nutrition policy so frequently—we’re about to do our next menu assessment to keep up with the program. (ECEC, Director)

Most participants from both groups emphasized that without a coordinated, multi-faceted approach across policy, workforce, family, and government support, nutrition implementation efforts would remain fragmented.

## DISCUSSION

The findings of this study provide insight into two key areas central to the study’s aim. These focus on stakeholders’ perspectives on the role and purpose of CBNPs in early childhood settings, and the factors that influence how these policies are implemented and supported in practice.

Participants in this study emphasized the importance of having CBNPs as a foundation for promoting healthy food environments in ECEC settings. However, they also highlighted that the effectiveness of these policies depends on how they are structured. Participants consistently advocated for policies that were clear, evidence based, and practical, yet adaptable enough to accommodate local needs and service-level variation. They also expressed a preference for policies that were structured and offered clear guidance and expectations without being overly rigid. While they valued alignment with national nutrition standards, many emphasized the need for policies that could be adapted to the context of their individual service.

This reflects broader advocacy for practical policy design where tools and resources are both evidence based and practical for frontline implementation, particularly in diverse, resource-variable ECEC settings (Kirkegaard *et al*. 2024). Future research and policy development should explore how flexibility can be built into CBNPs without compromising clarity or nutritional integrity. This aligns with previous research showing that while structured nutrition policies are associated with improved food provision and child dietary intake (Neelon and Briley 2011, Bell and Golley 2015, Benjamin-Neelon 2018, Matwiejczyk *et al*. 2018, Wolfenden *et al*. 2020), overly prescriptive guidelines may reduce feasibility and staff engagement, particularly in contexts where staff face competing demands and have limited nutrition training (Cumming 2017, Hollar *et al*. 2018, Grant *et al*. 2019, Thorpe *et al*. 2021, Kirkegaard *et al*. 2024). Both service- and agency-level participants in this study described instances where overly technical or nutrient-specific policy language became a barrier, particularly when staff lacked formal nutrition training. For example, educators found it difficult to interpret serving sizes or meet strict sodium limits when planning menus or communicating expectations to families. While current literature highlights the importance of using accessible and user-friendly language in early childhood nutrition policies (Kirkegaard *et al*. 2024), our findings further suggest that centre-based policies may benefit from adopting food-based, rather than nutrient-based, guidelines to improve clarity and uptake among non-specialist staff.

A related tension emerged between comprehensiveness and feasibility of a CBNP. While agency-level participants were more likely to emphasize the importance of detailed policies that align with national dietary guidelines, service-level participants tended to prioritize brevity, clarity, and adaptability to their specific contexts. However, both groups acknowledged the need for policies that were evidence-based and also practical to implement. This shared recognition highlights the importance of designing policy assessment tools that consider both components. For instance, while a tool such as the Wellness Child Care Assessment Tool (WellCCAT) is designed to assess comprehensiveness of CBNPs, future adaptations may benefit from considering factors such as readability, length, and language accessibility, particularly if intended for formative use by ECEC services themselves (Falbe *et al*. 2011).

Participants also raised concerns about prescriptive policies not accounting for differences in service size, budget, and community needs. For example, several participants working in lower-income areas reported difficulty meeting policy standards due to limited food budgets or access to fresh produce. This supports findings by Thorpe *et al*. (2020), who argue that universal policy frameworks may exacerbate inequalities if they do not account for service-level diversity (Thorpe *et al*. 2020). Without built-in flexibility, even well-intentioned policies can create additional pressures for educators who are already stretched across multiple responsibilities (Thorpe *et al*. 2021). In this context, external stakeholders could play a key role in helping services develop or adapt nutrition policies that reflect their unique contexts. This support may include assisting centres to review or tailor their policies to remain evidence informed while also being practical and achievable. While the existing national framework allows for local flexibility, clearer guidance and external input could help services navigate this process more confidently and equitably.

Ultimately, these findings regarding policy design signify that CBNPs should reflect nutritional standards and also anticipate the realities of the environments in which they will be enacted. Ensuring that policies are co-developed with service-level input, tested for usability, and supported by accompanying resources may increase both adherence and sustainability (Matwiejczyk *et al*. 2018, Wolfenden *et al*. 2020, Chan *et al*. 2025).

In line with the second research question, the findings also shed light on the multi-level factors that influence CBNP implementation and shape nutrition environments in practice. While policy design was viewed as foundational, participants consistently described service-level implementation as the point at which nutrition policies either succeed or stall. Both participant groups emphasized that CBNPs are only as effective as the people responsible for enacting them, highlighting the need for stronger engagement with families, better collaboration across roles, and meaningful recognition of employee efforts.

Family engagement was described as particularly important for reinforcing healthy eating behaviours beyond the ECEC environment. Several participants noted that when families were engaged through shared discussions, newsletters, or nutrition workshops, children were more likely to try new foods and continue healthy practices at home. Conversely, a lack of family involvement often resulted in mixed messages about food, which could undermine nutrition education efforts within the service. This finding aligns with research highlighting the importance of parent–educator collaboration in shaping early dietary habits, particularly in the context of food preference development and mealtime behaviours (Sisson *et al*. 2017, Scaglioni *et al*. 2018, Wallace *et al*. 2020). In this study, engagement strategies were also perceived to be the most effective when tailored to family contexts, and when they promoted shared ownership rather than imposing expectations.

Collaboration across roles within ECEC services also emerged as an implementation enabler. Educators, cooks, and directors were described as working more effectively when policies were developed collaboratively, with clear communication about expectations and shared responsibilities. In services where these roles were siloed, nutrition policies were more likely to be overlooked or inconsistently applied. In particular, participants noted that ECEC cooks/chefs were often excluded from key conversations about children’s dietary needs, food provision policy decisions, and access to professional development. This lack of integration was seen to limit their understanding of the broader educational goals of nutrition, potentially resulting in a task-focused rather than child-centred approach to food provision. Several participants advocated for greater inclusion of ECEC cooks/chefs in team meetings and training sessions to support more cohesive policy implementation. These findings are consistent with literature highlighting the importance of a ‘whole-of-service’ approach to health promotion, where shared accountability and mutual respect across roles contribute to stronger policy enactment (Weiss *et al*. 2016, Green *et al*. 2020).

Whole-of-service collaboration, however, was said to often be challenged by time pressures, staff shortages, and difficulties maintaining consistent communication across roles. Some ECEC educators described challenges in managing nutrition-related tasks alongside their existing responsibilities, particularly in environments where collaboration or support was limited. This reflects existing evidence on the emotional and cognitive labour of ECEC educators, and persistent concerns about the increasing workload and competing demands placed on ECEC professionals (Cumming 2017, McDonald *et al*. 2018). Without sufficient staffing and time allocation, even motivated ECEC employees may struggle to meaningfully engage with CBNPs.

Participants also spoke about the value of child engagement, through diverse food-related activities, to strengthen food knowledge and exposure. Initiatives such as edible (food) gardens and cooking activities were described as effective in helping children build positive relationships with food. According to participants, these practices fostered curiosity, confidence, and a willingness to try new foods, which are outcomes that align with the broader goals of CBNPs. While these practices were not always explicitly linked to CBNPs, they were seen as key components of a supportive food environment and opportunities to embed food education. These findings are supported by research demonstrating that experiential food education enhances children’s food acceptance and dietary behaviours (Johannessen *et al*. 2018, Kähkönen *et al*. 2018, Varman *et al*. 2021). Although the NQS already provides scope for such practices, these findings suggest CBNPs could more explicitly promote or enable food-based learning activities as part of a comprehensive nutrition approach.

Overall, participants emphasized that service-level implementation of CBNPs required more than individual effort and instead depended on a coordinated, well-resourced approach that respected the expertise of educators, actively engaged families, and embedded nutrition into everyday practice in a way that was both enjoyable and sustainable. Participants also expressed a clear and consistent need for stronger, better-coordinated government support to enable the effective implementation of CBNPs. While most participants supported the intent of existing national dietary guidelines and policy frameworks, they described a significant gap between expectations and the level of practical support available to services, particularly in relation to funding, training, and access to clear, actionable resources.

Many participants reported that current government guidance is fragmented, inconsistent across jurisdictions, and often inaccessible to those working on the ground. ECEC employees described having to locate information from multiple sources, which made it difficult to interpret or apply nutrition requirements or policies in daily practice. This reflects broader concerns about the accessibility of nutrition standards and the need for streamlined, well-communicated resources tailored specifically to ECEC settings (Seward *et al*. 2017, Mozaffarian *et al*. 2018, Spence *et al*. 2020). Participants recommended that governments play a greater role in curating and disseminating simplified, service-ready materials, such as template policies and visual menu planning tools, to reduce reliance on individual educators to interpret complex policy documents.

Beyond guidance, funding was identified as a critical enabler of quality nutrition provision. Participants emphasized that in services where meals were provided, food budgets often limited what could be served. Some described per-child food allocations as low as $1 per day forcing reliance on low-cost staples such as pasta and toast, with limited inclusion of vegetables, fruits, or lean proteins. This finding is supported by previous research highlighting the direct relationship between food budgets and nutritional quality in ECEC services (Dev *et al*. 2017, Sambell *et al*. 2020, Fjaera *et al*. 2022, Searle *et al*. 2024). Such financial constraints were also seen to hinder the practical implementation of CBNPs, especially where policies included expectations around food variety, quality, and portion sizes. Participants advocated for increased government investment to subsidize healthy food provision, particularly in ECEC services operating in disadvantaged communities or with high proportions of food-insecure families, to enable equitable access to nutritious meals. This was considered a key responsibility of government bodies as a mechanism to reduce health disparities from early childhood.

Professional development was also highlighted as a priority for government investment. While some stakeholders acknowledged the value of state-based programmes, others noted these were not consistently available across all states or did not include sufficient depth on nutrition-specific topics. Participants expressed a need for structured, ongoing training particularly for ECEC educators and cooks responsible for implementing nutrition policies. Such training would need to go beyond general awareness and build confidence in applying nutrition principles in diverse, real-world settings. This aligns with previous findings suggesting that many ECEC professionals feel underprepared to deliver nutrition education or implement nutrition guidelines, particularly when they have no formal training in the area (Sisson *et al*. 2017, Wallace *et al*. 2017, Love *et al*. 2020, Omdal and Roland 2020, Helland *et al*. 2023).

In addition to funding and training, participants described the importance of aligning efforts across sectors to support cohesive implementation. It was suggested that rather than operating in silos, government agencies, health departments, and education systems should work collaboratively to ensure consistency between national dietary guidelines, accreditation systems, and local-level nutrition programmes. Participants proposed examples of cross-sector collaboration, including agency support for menu development, shared training initiatives, and efforts to engage families in promoting healthy eating. These collaborations were viewed as essential for bridging the gap between policy intent and practical implementation. Importantly, participants emphasized that such cross-sector efforts must include genuine collaboration with ECEC services themselves, recognizing their expertise and central role in shaping and implementing contextually appropriate solutions. This aligns with calls in the Australian literature for co-design approaches that are grounded in service-level realities and led in partnership with the sector (Elford *et al*. 2025, Natale *et al*. 2025, Wong *et al*. 2025).

These findings point to a need for clearer communication, collaboration, and shared ownership, in which responsibility for both policy development and implementation is distributed across sectors, including policymakers, health professionals, and ECEC employees. Strengthening these partnerships could involve the co-design of policies and support tools, shared implementation frameworks, and integrated monitoring and feedback systems. For example, including ECEC representatives in government-led working groups on child nutrition policy could ensure that policy recommendations are grounded in service realities. Likewise, health professionals could be formally engaged to support ECEC services through mentoring, resource development, or training delivery, to provide consistent, evidence-based support rather than *ad hoc* engagement.

Ultimately, these findings demonstrate the need for a system-level approach to CBNP implementation—one in which services are not left to create, interpret and implement policies in isolation but are supported by adequate resourcing and a coordinated network of government agencies, training bodies, and health sector partners. Without this alignment, even well-designed CBNPs risk remaining aspirational documents rather than tools for meaningful change.

### Strengths and limitations

To our knowledge, this is the first Australian study to explore CBNPs in ECEC settings through the lens of both policy developers and on-the-ground practitioners, providing a valuable contribution to the field by highlighting real-world challenges and enablers from multiple levels of influence. A key strength of this study is its inclusion of diverse stakeholders, providing a comprehensive understanding of both service- and agency-level perspectives.

The national reach of the agency-level sample further strengthened the findings by enabling the identification of context-specific and cross-cutting barriers to CBNP implementation across Australia. However, the service-level sample was primarily from Victoria and NSW, which may not fully reflect systemic challenges in other states and territories. Given that policy support structures and implementation contexts vary across jurisdictions, future research should seek broader geographic representation of service-level perspectives to ensure findings are nationally applicable. While the findings offer rich insight into the perspectives of those interviewed, they are not intended to be statistically generalizable. Instead, they may be transferable to similar settings where policy and service conditions align. As with all qualitative research, context is central to interpretation, and transferability will depend on the degree of similarity to other implementation environments (Lincoln and Guba 1985, Liamputtong 2019, Enworo 2023). Additionally, recruitment was limited to those who responded and consented. Therefore, findings may not be fully representative of all perspectives across the ECEC and health sectors. The sample size of service-level participants, while sufficient for qualitative analysis, may also limit the generalizability of the findings. Future research could benefit from a larger, more diverse sample to explore whether the themes identified in this study hold across a broader reach of ECEC services.

Researcher subjectivity is another consideration in qualitative work. While every effort was made to enhance credibility through collaborative coding, regular peer debriefing, and reflexivity, some degree of interpretive bias may remain.

There were also some limitations in the agency-level sample; for instance, the affiliation of several stakeholders was not always clearly identifiable, limiting claims of national representativeness.

Future research could build on these findings by capturing greater diversity within stakeholder groups, including broader jurisdictional, socioeconomic, and service-type representation, as well as variation in food provision models. Doing so strengthens the evidence base and provides a more comprehensive foundation for informing future policy and practice.

## CONCLUSION

Our investigation into stakeholder perspectives highlights the complexities of fostering optimal nutrition environments in early childhood education settings. These findings reinforce the multi-faceted nature of CBNP implementation, emphasizing the need for collaborative and adaptable approaches that integrate evidence-based practices with the practical realities of ECEC settings. Strengthening partnerships between policymakers, health professionals, and ECEC employees is essential to co-design policies, resources, and training that reflect both nutritional standards and implementation feasibility. Furthermore, cross-sector alignment in health, education and community services is needed to coordinate guidance, professional development, and programme delivery that is consistent and better meets the needs of diverse ECEC contexts. Together, these actions can support the creation of nutrition environments that meet established standards while remaining sustainable, equitable, and responsive to the communities they serve. This approach lays a stronger foundation to support ECEC sector stakeholders in promoting lifelong healthy eating behaviours among young children.

## Supplementary Material

daaf165_Supplementary_Data

## Data Availability

The data underlying this article cannot be shared publicly due to its qualitative nature and to maintain the anonymity of study stakeholders. The data will be shared on reasonable request with the corresponding author.
