# Supplementary material for: From policy to plate: stakeholder perspectives on nutrition policy in Australian early childhood education environments
Source: Health Promot Int. 2025 Oct 3;40(5):daaf165. doi: 10.1093/heapro/daaf165 (PMC12491997; doi:10.1093/heapro/daaf165)
Supplement: daaf165_Supplementary_Data [file daaf165_supplementary_data.docx]

**Supplementary File 1. Interview Inquiry Logic**

| **Inquiry Logic** | **Interview questions** | **Probing questions** |
| --- | --- | --- |
| ***Agency Stakeholders*** | | |
| Understanding stakeholders' roles and responsibilities related to nutrition and policy within their organisations. | Can you describe your role within your organisation and its relevance to early childhood nutrition or nutrition policy in childcare? | Could you provide more detail about your responsibilities within your organisation and how they intersect with early childhood nutrition or nutrition policy in ECEC settings?  How do you see your role contributing to ensuring the nutritional needs of young children are met? |
| Assessing stakeholders' knowledge and expertise in the field of early childhood nutrition and policy. | What experience do you have with early childhood nutrition and/or policy? | Can you share any specific experiences or instances where you've been involved in early childhood nutrition initiatives or policy development?  How have these experiences shaped your understanding of the importance of nutrition in childcare settings? |
| Identifying perceived challenges in implementing nutrition policies within childcare centers. | What do you perceive as the primary challenge in Long Day Care centres concerning nutrition and policy in the ECEC setting? | Are there any specific factors or issues that you believe contribute to this challenge?  How do you think these challenges impact the overall well-being of children in childcare? |
| ***ECEC Stakeholders*** | | |
| This question aims to gather information about the participant's specific role within the childcare center. Understanding the participant's role will provide context for their responses to subsequent questions regarding nutrition policy and challenges | I see from your survey you indicated you work as [response] (i.e. chef/educator/director). Can you tell me about what this role involves? | Could you elaborate on some of the key responsibilities you have in your role as [Chef/Educator/Supervisor]?  How do these responsibilities relate to food provision and nutrition? |
| This question seeks to explore the participant's familiarity and engagement with nutrition policy within the childcare setting.  Responses will provide insights into the participant's level of involvement in policy development, implementation, or adherence. | What is your experience or involvement with nutrition policy? | Can you share any specific instances where you've been directly involved in shaping or implementing nutrition policies within your childcare center? |
| This question aims to uncover the participant's firsthand experiences with policy changes related to food provision in childcare centers. By eliciting specific examples, the researcher can gain insights into the impact of policy changes on daily operations and practices | During your work career, what policy changes have you experienced at your childcare center in relation to the provision of food to children? | What changes have you observed or been part of regarding food-related policies during your time working in childcare?  How did these changes impact your daily routines or interactions with children? |
| This question is designed to identify the participant's perceptions of the primary challenges associated with nutrition and policy in Long Day Care centers.  Responses will highlight key areas of concern or difficulty that may require attention or intervention. | What do you see as the biggest challenge in Long Day Care centers when you think about nutrition and policy in the setting? | Could you expand on why you perceive nutrition and policy as challenging in Long Day Care centers?  Are there any specific obstacles or barriers you frequently encounter? |
| This question aims to solicit the participant's perspective on support mechanisms that would enhance their effectiveness and well-being in their role.  Responses will provide valuable insights into the types of support strategies that may be beneficial for childcare center staff. | What is one way or support strategy that would make you feel more supported in your role? | If you had the opportunity to introduce a new support strategy in your role, what would it look like?  How do you think it would improve your ability to address nutrition-related challenges effectively? |
| ***Survey Response Exploration*** |  |  |
| *Nutrition Policy:*  Exploring stakeholders' opinions and insights regarding the importance and implementation of nutrition policies within ECEC centres | You indicated that it was important for childcare centers to have nutrition policies outlining the types of food served. Could you please elaborate on why you chose this answer? | You mentioned reasons for the difficulties in implementing these policies. What solutions or changes do you suggest to improve their implementation? |
| *Nutrition Education:*  Delving into stakeholders' perspectives on the significance and implementation of nutrition education within ECEC centres. | You expressed the importance of including Nutrition Education in centre policies. Could you please elaborate on the reasons behind your choice?  Nutrition Education Matrix:  If any unique or intriguing selections are made (listed prior to the interview), stakeholders will be asked to elaborate.  "You selected [response] for item X. Could you please explain one reason behind your choice?"  Additional Element Identified for the Domain: Y/N (to be circled before the interview for reference)  If any additional item is listed that lacks self-explanation, it will be briefly discussed as it may warrant inclusion in the revised tool.  Additional Comments Section:  Stakeholders will be requested to elaborate on any additional comments provided. These comments are expected to pertain to a particular theme, such as staff education, resources, or socioeconomic factors. Themes will be identified in advance to facilitate detailed discussion during the interview. | For instance, what factors influenced your decision?  Could you share any experiences or instances where you've seen the impact of nutrition education on children or caregivers within your centre?  How did these experiences influence your perspective on the importance of including nutrition education in policies?  In your opinion, how do you think nutrition education contributes to the overall well-being and development of children in ECEC settings?  Can you provide examples that illustrate this impact? |
| *Nutrition Standards*  Investigating stakeholders' views on the importance and integration of nutrition standards within ECEC centre policies. | You expressed that it was "X important" for centre policies to incorporate Nutrition Standards. Could you please elaborate on the reasons behind your choice?  Nutrition Standards Matrix: Yes/No  If any uncommon or noteworthy selections are made (listed before the interview), stakeholders will be prompted to provide an explanation.  For instance, you indicated [response] for item X. Could you please explain one reason behind your choice?  Additional Element Identified for the Domain: Y/N (to be circled before the interview for reference)  If any additional item is listed that lacks self-explanation, it will be briefly discussed as it may warrant inclusion in the revised tool.  Additional Comments Section:  Stakeholders will be requested to elaborate on any additional comments provided. | What are some of the factors that influenced your selection of this answer?  What specific benefits or outcomes do you anticipate from integrating nutrition standards into centre policies?  Can you share any experiences or situations where you've observed the impact of nutrition standards on the dietary habits or health outcomes of children in ECEC settings?  How did these experiences influence your perspective on the importance of including nutrition standards in policies?  What challenges do you think there are in implementing nutrition standards within ECEC centres? |
| *Nutrition Promotion:*  Examining stakeholders' thoughts and perspectives on the role and significance of nutrition promotion within ECEC policies. | You expressed that including Nutrition Promotion in centre policies was deemed "X important." Could you please elaborate on the reasons behind your choice?  Nutrition Promotion Matrix: Y/N  If any uncommon or intriguing choices are noted (listed before the interview), the participant will be prompted to provide an explanation.  For instance, if you indicated [response] for item X, could you please explain one reason behind your selection?  Additional Element Identified for the Domain: Y/N (to be circled before the interview for reference)  If any additional item is listed that lacks self-explanation, it will be briefly discussed as it may warrant inclusion in the revised tool.  Additional Comments Section:  Stakeholders will be requested to elaborate on any additional comments provided. | For instance, what are some of the factors that led you to select this answer?  What do you think are the main challenges in implementing Nutrition Promotion in ECEC centres?  Any ideas on how to overcome these challenges?  In your opinion, what strategies or approaches are most effective in promoting healthy eating habits among young children ECEC centres? |
| *Nutrition Evaluation/Communication:*  Understanding stakeholders' perceptions regarding the importance and implementation of nutrition evaluation and communication within ECEC policies. | You noted the significance of incorporating Nutrition Evaluation/Communication into center policies. Could you please expand on the rationale behind your choice?  Nutrition Evaluation Matrix: Y/N  If any unconventional or noteworthy selections are made (to be listed prior to the interview), you will be prompted to elaborate.  You indicated [response] for item X. Could you please explain one reason behind your selection?  Additional Element Identified for the Domain: Y/N (to be circled before the interview for reference)  If any additional item is listed that lacks self-explanation, it will be briefly discussed as it may warrant inclusion in the revised tool.  Additional Comments Section:  Stakeholders will be requested to elaborate on any additional comments provided. | For example, what are some of the reasons you provided this response?  Are there any specific challenges you've encountered related to policy evaluation in your centre? How have you addressed or overcome these challenges?  Can you provide an example of how effective nutrition evaluation or communication has positively impacted children's dietary habits or overall well-being in your centre?  What strategies do you think are most effective in communicating nutrition-related information to both children and their families in childcare settings? |
